# Annual and Analytical Cyclopædia of Practical Medicine

**Published:** 1898-09

**Authors:** 


**Annual and Analytical Cyclopædia of Practical Medicine.** Vol. I. Philadelphia: The F. A. Davis Company. 1898.

After many years of careful consideration, Dr. Sajous, the editor of the well-known *Annual of the Universal Medical Sciences*, has worked out a satisfactory solution of the problem how to combine the features of a text-book with the *Annual* as it has hitherto appeared. This, the first volume of a new publication, has an alphabetical arrangement of the subjects, comprising all the practical topics of medicine and surgery and the clinical application of therapeutics from A to Bright's Disease. Each subject includes in large type, a concise statement of the generally accepted methods in vogue while in small type on the same page can be found the opinions of well-known authorities on whatever may be debatable. The work will appear at the approximate rate of one volume each six months, the whole series being completed in three years.

During this long period needful to complete the alphabet, it is proposed to supply to subscribers a monthly cyclopædia from A to Z, so that every subscriber will have a complete synopsis of the latest journal literature to reinforce his system of reference. The aim of Dr. Sajous and his staff of one hundred associate editors is designed to accomplish two things: (1) To give a satisfactory statement of what may be safely relied upon as the best general method of treatment in any given case; (2) To combine with this a means of practically utilising the discussion by the leading medical authorities of the world, which may in any degree modify present established methods. The work they have undertaken is enormous, and it is exceedingly well done. Hitherto the *Annual* had made for itself a place among writers, teachers, and investigators: now it is designed to help the hard-worked practitioner in the accomplishment of his duty, as he will find there suggestions innumerable,—not only those usually considered in classic works, but also all the practical points worth recording found in the literature of the last decade. Nothing has been spared to make the work useful to its readers.

